# Decay of parasite community similarity with host phylogenetic and geographic distances among deep-sea fish (grenadiers)

**DOI:** 10.1017/S0031182022001251

**Published:** 2022-11

**Authors:** Xuhong Chai, Jerusha Bennett, Robert Poulin

**Affiliations:** Department of Zoology, University of Otago, P.O. Box 56, Dunedin 9054, New Zealand

**Keywords:** Community composition, deep-sea fish, distance decay, geography, helminth diversity, Macrouridae, parasite assemblages, phylogenetic relatedness, rattails

## Abstract

Although parasite community studies are growing in numbers, our understanding of which macro-ecological and evolutionary processes have shaped parasite communities is still based on a narrow range of host–parasite systems. The present study assessed the diversity and endoparasite species composition in New Zealand deep-sea fish (grenadiers, family Macrouridae), and tested the effects of host phylogeny and geography on the structure of endoparasite communities using a distance decay framework. We found that grenadiers from the Chatham Rise harboured a surprisingly high diversity of digeneans, cestodes and nematodes, with different species of grenadiers having different parasite assemblages. Our results demonstrate that community similarity based on the presence/absence of parasites was only affected by the phylogenetic relatedness among grenadier species. In contrast, both phylogenetic distance among grenadiers (measured as the number of base-pair differences of DNA sequences) and geographic distance between sample locations influenced the similarity of parasite communities based on the parasites' prevalence and mean abundance. Our key findings highlight the significant effect of deep-sea host phylogeny in shaping their parasite assemblages, a factor previously neglected in studies of parasite communities in deep-sea systems.

## Graphical abstract

**Figure:**
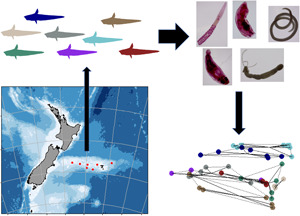

## Introduction

Due to the biodiverse and ubiquitous nature of parasites (Poulin and Morand, 2000; Poulin, 2014), there is growing appreciation of the roles they play in shaping our knowledge of community ecology. In marine systems, fish parasites undoubtedly account for a significant component of marine biodiversity, and significant advances have been achieved in our understanding of the composition and structure of their assemblages in coastal marine fish (Munoz *et al*., 2006; Marques *et al*., 2011; Braicovich and Timi, 2015). However, primarily due to the limited access to deep-sea organisms, few studies focus on what shapes parasite community assemblages of deep-sea fish (Dallarés *et al*., 2016; Espínola-Novelo *et al*., 2018). Considering that deep-sea fish account for approximately 30% of all known fish families, and many are considered ‘primitive’ (Weitzman, 1997), investigation of their parasite assemblages can provide insights into host and parasite species radiation between and within marine environments (Bray, 2020).

The exponential decay of similarity in species assemblages as the geographical distance between localities increases has been acknowledged in the field of biogeography as a universal law, and an important pattern of community composition at a macroecological scale (Morlon *et al*., 2008). The ecological drivers of the distance decay in similarity include changing environmental factors across space and dispersal constraints (Nekola and White, 1999). Most distance-decay studies in parasitology have been conducted in terrestrial and freshwater environments, as these habitats are usually thought to be more fragmented than marine ones, leading to a higher expected rate of similarity decay with increasing distance (Poulin, 2003). Decay of similarity over distance has also been detected at large geographical scales in parasite communities of marine fish (e.g. Oliva and Gonzalez, 2005; Braicovich *et al*., 2017). However, in other studies, this was only observed at the individual host level using parasite abundance data, possibly due to local environmental factors mediating the availability of intermediate hosts as prey items for fish hosts (Pérez-del-Olmo *et al*., 2009).

Because both host ecology and phylogeny determine parasite assemblages (Brooks, 1980; Holmes and Price, 1980), while an exponential decay of similarity in parasite community composition occurs on geographical scales, a similar process should be apparent in phylogenetic space. If an ancestral parasite and an associated ancestral host form a long evolutionary relationship, the parasite's descendants should be associated with the host's descendants (Brooks, 1988). Under this scenario, closely related host species can harbour similar parasite faunas which are inherited from their most recent common ancestor. If some parasite species shared between closely related hosts have been acquired more recently rather than inherited from a common ancestor, those new parasites should reflect differences in the ecology, physiology and immunology between different host species, largely determined by their phylogenetic relatedness (Peterson *et al*., 1999). The decay of similarity in parasite faunas as a function of increasing host phylogenetic distance has so far only been tested by Seifertova *et al*. (2008) and Poulin (2010). Their findings suggest that both geographic and phylogenetic distance among hosts can shape parasite species composition, resulting from a combination of evolutionary and ecological processes.

The association between deep-sea fish and their parasites provides a unique system to test the impact of host phylogenetic relatedness and geographical distances on parasite assemblages. The deep-sea environment exerts stronger abiotic constraints (lack of sunlight and nutrients, high pressure and low temperature) than coastal marine areas (Rex and Etter, 2010); the dispersal of deep-sea parasites with complex life cycles is therefore likely to be more dependent on their fish hosts than in shallow coastal regions. Therefore, deep-sea parasite assemblages may be more prone to the influence of both host phylogeny and geography. However, fish-feeding ecology is seen as the main determinant of deep-sea parasite assemblages, with potential influences of host phylogeny being negligible (see discussion in Palm and Klimpel, 2008). Most geographical comparisons just draw broad conclusions from contrasts between different large-scale geological regions (Bray, 2020), rather than quantifying the impact of distance between fish hosts on a smaller scale, as pointed out by Campbell (1990).

In this study, the endoparasites of New Zealand (NZ) grenadiers (or rattails, family Macrouridae) from the Chatham Rise were investigated to assess their diversity, and evaluate the contribution of host phylogenetic relatedness and geographical distance to the interspecific similarity of their parasite assemblages in a distance-decay context. NZ grenadiers and their parasites are a great model system for parasite community study, since grenadiers are very speciose and abundant on the Chatham Rise, providing an opportunity to compare the effect of host phylogeny and geography on their parasite communities in a local scale (Stevens *et al*., 2020). We hypothesize that both phylogenetic relatedness and geographical distance among host species affect their parasite assemblages. Phylogenetic distance (number of base-pair differences of DNA sequences) measures the relatedness of all pairs of host species, whereas geographic distances between pairs of sampling sites measure potential ecological differences between two sites. Because phylogeny and ecology can affect parasite assemblages in different ways (Ñacari and Oliva, 2016), we predict a decay of parasite presence/absence similarity as the pairwise phylogenetic distance between grenadier species increases, and a decay of similarity based on parasite prevalence and mean abundance as both pairwise phylogenetic and geographic distances increase. This study not only expands our knowledge of parasites of NZ grenadiers by providing new host and geographic records for some parasites, but also provides insights into grenadier ecology, such as their feeding ecology, since parasite assemblages indirectly reflect the ecological characteristics of their hosts (Campbell *et al*., 1980; Palm and Klimpel, 2008). Furthermore, by investigating the impact of host phylogenetic relatedness and geographic distance in a quantitative way, this study provides insights into the effects of evolutionary history and biogeography on the formation of parasite communities in this unique deep-sea system.

## Materials and methods

### Sampling of deep-sea fish hosts and their parasites

**Fig. 1.**
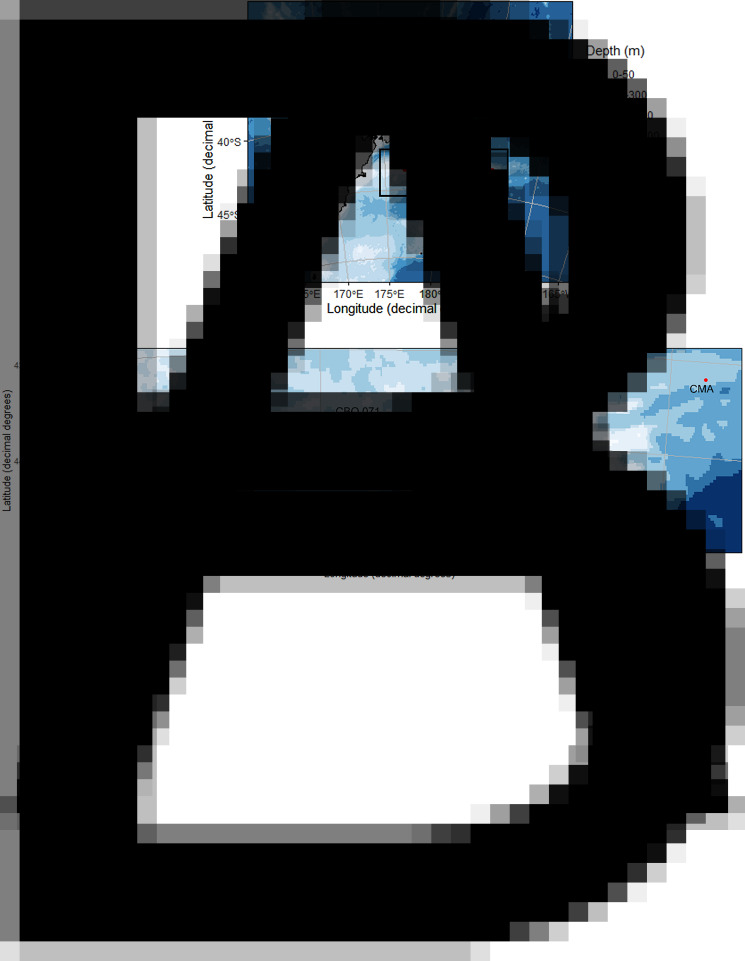
Grenadier sampling locations on the Chatham Rise, New Zealand. (A) Chatham Rise (black rectangle) relative to New Zealand, (B) Close-up of trawling locations along the Chatham Rise. Trawling sites where grenadiers were collected are marked by red dots. Species codes: CMA: *Coelorinchus matamua* (*n* = 3); CFA: *C. fasciatus* (*n* = 5); CBI: *C. biclinozonalis* (*n* = 6); CBO: *C. bollonsi* (*n* = 8); CAS: *C. aspercephalus* (*n* = 7); BJA: *Medobius antipodum* (*n* = 6); JAV: *Lepidorhynchus denticulatus* (*n* = 4); CSU: *Coryphaenoides subserrulatus* (*n* = 7). A single CBO specimen – CBO 071 – was caught in a different site in trawl 071, while all other CBO specimens were caught in trawl 057.

Grenadiers and their parasites were collected during a National Institute of Water and Atmospheric Research (NIWA) trawl survey along the Chatham Rise between January and February 2020 (see trawl depth of fish collection in Table 1). The Chatham Rise is a submarine ridge about 1000 km from the east coast of the South Island of NZ (Stevens and Dunn, 2011). Samples contained endoparasites collected from 52 grenadier individuals (with non-everted stomachs) of 8 different species, including 5 species within the genus *Coelorinchus* and 1 each in the genera *Coryphaenoides*, *Mesobius* and *Lepidorhynchus*, all in the family Macrouridae (Fig. 1, Table 1). Endohelminths were collected from host intestinal organs and body cavity, and pieces of host muscle tissue from 6 species of grenadiers (all except for *Coelorinchus matamua* and *Lepidorhynchus denticulatus)* were collected. All samples were preserved in 70% EtOH.

**Table 1.** Information regarding grenadier samples collected on the Chatham Rise, New Zealand

Grenadier species	Host code	Latitude	Longitude	*n*	Total length Mean (cm) ± s.d.	Weight Mean (g) ± s.d.	Sex	Trawl Depth range (m)
*Coelorinchus matamua*	CMA	43°12′S	174°30′W	3	44.4 ± 6.1	385.0 ± 192.2	2F, 1 M	868–883
*Coelorinchus fasciatus*	CFA	44°18′S	179°48′W	5	30.5 ± 0.8	126.0 ± 17.5	5F	878–882
*Coelorinchus biclinozonalis*	CBI	43°30′S	177°00′E	6	41.6 ± 5.4	322.5 ± 144.4	6F	260–279
*Coelorinchus bollonsi*	CBO	43°48′S	179°42′W	1	51.9 ± 0.0	750.0 ± 0.0	1F	379–383
		43°42′S	177°36′W	7	51.4 ± 5.6	877.1 ± 205.7	7F	390–396
*Coelorinchus aspercephalus*	CAS	43°42′S	177°36′W	7	33.1 ± 1.9	230.0 ± 46.5	7F	390–396
*Mesobius antipodum*	BJA	44°30′S	178°30′W	12	44.9 ± 5.5	362.9 ± 119.3	3F, 9 M	975–998
*Lepidorhynchus denticulatus*	JAV	43°42′S	178°36′E	4	NA	NA	NA	392–398
*Coryphaenoides subserrulatus*	CSU	44°30′S	178°30′W	7	NA	NA	NA	975–998

The latitude and longitude coordinates represent the proximate medium point of the start and end for each of the trawling positions. Sex: F – female, M – male.

### Molecular study for parasites and their fish hosts

Molecular tools were the main method used for helminth species identification, with complementary morphological identification for some digeneans. Representatives of each parasite and host species (for the purpose of calculating phylogenetic distance, see following distance-decay method section) were chosen for PCR. DNA was extracted using the Chelex extraction method (Walsh *et al*., 1991). We used primers T16 and T30 from Harper and Saunders (2001) to resolve a partial fragment of the 28S gene for digeneans (~600 bp length) and cestodes (~700 bp length) following the PCR conditions from Presswell and Bennett (2020). For nematodes, a partial fragment of the 18S gene was resolved (~800 bp length) using primers Nem18SF and Nem18SR from Wood *et al*. (2013) following PCR conditions of Prosser *et al*. (2013). For grenadier hosts, 585 bp length of the *cox*1 gene was resolved using FishF2, FishR1 and FishR2 primers (Ward *et al*., 2005) following PCR conditions of Ward *et al*. (2005). PCR products were cleaned using ExoSAP-IT™ Express PCR Product Cleanup Reagent (USB Corporation, Cleveland, OH, USA) following the manufacturer's instructions. Sanger sequencing by capillary electrophoresis was performed by the Genetic Analysis Service, Department of Anatomy, University of Otago (Dunedin, NZ). Sequences were imported into Geneious Prime 2022.0.2 (https://www.geneious.com), trimmed using default settings and manually edited for ambiguous base calls. Each sequence was uploaded to the BLAST online search tool (www.ncbi.nlm.nih.gov) (Altschul *et al*., 1990, 1997) to confirm their identity to the lowest taxonomic level possible. See supplementary material for BLAST results.

### Morphological identification for digeneans

Digenean species (all but 1 poorly preserved specimen) were identified using morphology where possible. Representative specimens were stained in acetic acid iron carmine, dehydrated in an ethanol series, cleared in clove oil and permanently mounted with Canada balsam for light microscopy. Morphological keys (Blend *et al*., 2000; Gibson *et al*., 2002; Bray *et al*., 2013) and original species descriptions (Manter, 1925; Yamaguti, 1938; Gibson and Bray, 1986; Bray and Jones, 1993) were used to identify parasites to the lowest taxonomic level possible.

### Parasite community descriptors

Analysis of parasite species composition among different fish hosts was conducted at both component community and Infracommunity levels. The presence/absence of particular parasite species, total abundance and species richness of parasites at the infracommunity level, as well as the presence/absence, prevalence and mean abundance of parasite species at the component community level were calculated following Bush *et al*. (1997). When necessary, because not all individual worms were analysed genetically, parasite species that were indistinguishable based on morphology were treated as the same taxon for all subsequent analyses.

### Visualizing parasite assemblages among different grenadier species

Parasite species composition in different host species was visualized by using community descriptor data to construct classical multidimensional scaling (MDS) plots and dendrograms produced from hierarchical agglomerative clustering (HAC) analysis (with the average linkage method). Both MDS plots and dendrograms were created at the infracommunity level with presence/absence and total abundance data. In addition, MDS plots and dendrograms were also computed for infracommunity presence/absence of digeneans, cestodes and nematodes separately. At the component community level, only a dendrogram was produced with presence/absence, prevalence and mean abundance data. Dendrograms for the presence/absence of digeneans, cestodes and nematodes at the component community level were also produced. All statistical analyses were conducted in R version 2021.09.0 (R Core Team, 2021).

### Parasite assemblage analysis at the infracommunity level

Differences among parasite assemblages of different host species at the infracommunity level were tested with one-way permutational multivariate analysis of variance (PERMANOVA) on both presence/absence and total abundance of parasite species data. Using fish species as the fixed effect, the parasite community structures of each grenadier host were compared between grenadier species, with the fixed effect tested after 999 permutations. The Jaccard method was used to create the distance matrix with presence/absence of infracommunity data, while the Bray–Curtis method was used with total abundance data (Magurran, 1988).

PERMANOVA assumes homogeneity of variances, therefore, a distance-based dispersion test which uses the PERMDISP2 procedure from Anderson (2006) was conducted on the infracommunity data after the PERMANOVA. Both analysis of variance (Anova) and a Permutation test for homogeneity of multivariate dispersions (permutest) with 999 permutations were used to test for differences of dispersions (variances) between different groups (fish species).

To further investigate the differences in parasite assemblages among different species of grenadiers, pairwise PERMANOVA was used to compare the parasite species community structure between all pairs of grenadier species. The default method for adjusted *P* value, ‘bonferroni’, was used in the analysis since it is the most conservative correction. Fish species were used as the factor in comparisons, and it was tested after 999 permutations.

### Species richness variation with fish length and fish species

Host size and sex were also tested against parasite community descriptors to rule out their potential impact. Fish length correlated with weight (simple linear model: *P* < 0.001, *R*^2^ = 0.84), but did not differ between sexes (Anova: *P* = 0.57, *F*_1,39_ = 0.33). Since fish sex was strongly biased towards females (only 10 out of 41 fish with sex data available were male, see Table 1), only fish length was included in the analysis. Two generalized linear models (GLMs) were used to test the effect of fish length (NB: only 6 out of 8 grenadier species had length data available, see Table 1) and fish species (as a categorical variable), on parasite species richness at the infracommunity level. Both GLMs used the ‘poisson’ as family. Dispersion checking was conducted by running another GLM assuming the data distribution was ‘negative binomial’ instead of ‘poisson’, then GLM models were ranked by AIC to select a better model.

### Parasite assemblage at component community level – distance decay analysis

Similarity in parasite species composition was analysed at the component community level. Jaccard similarity index was computed using qualitative community descriptors (presence/absence data), while Bray–Curtis similarity index was calculated with quantitative data (mean abundance and prevalence data) (Magurran, 1988).

As mentioned earlier, no host tissue was collected for *C. matamua* and *L. denticulatus*. Therefore, we downloaded the only available *cox1* sequence for *C. matamua* found on Genbank (Accession: MN123316) and 1 of the *cox1* sequences for *L. denticulatus* (Sample ID: BW-A13226) found in the Barcode of Life database (https://www.boldsystems.org/). These sequences were aligned with 1 *cox1* sequence we produced for each of the other grenadier species using the MAFFT alignment G-INS-I default algorithm (Katoh and Standley, 2013) in Geneious Prime 2022.0.2. All aligned sequences were trimmed to equal length (585 bp) and imported into MEGA11: Molecular Evolutionary Genetics Analysis version 11 (Tamura *et al*., 2021) to calculate the pairwise uncorrected p-distances based on the proportion of base pair differences among sequences.

Distance (in kilometres) between each pair of trawling locations on the Chatham Rise was calculated using their coordinate data with an online latitude/longitude distance calculator (https://www.nhc.noaa.gov/gccalc.shtml). The single *Coelorinchus bollonsi* caught at a different site was treated as though captured at the same location as its conspecifics.

Jaccard and Bray–Curtis similarity indices were log-transformed to meet the assumptions of normality for parametric testing. Three multiple linear regressions were separately performed on pairwise similarity indices computed by presence/absence, prevalence and mean abundance data, with the matching pairwise phylogenetic and geographic distance data as predictor variables, with the sample size equalling the number of pairwise comparisons.

To account for the non-independence of the data (the same fish species was used in multiple comparisons), all linear models were re-run with regression fitted with permutation tests with 999 permutations.

### Species accumulation curve

To estimate the adequacy of grenadier sample size in this study to assess parasite diversity in our system, the cumulative number of parasite species recovered was plotted against the cumulative number of grenadier individuals examined, including only those infected by parasites (Dove and Cribb, 2006). The 95% confidence interval was calculated by 1000 permutations.

## Results

The parasite fauna of grenadier hosts comprised a total of 35 parasite species (Table 2). A total of 984 parasites were obtained from 52 individual fish belonging to 8 species. All individuals in 7 host species were infected by parasites; for *Mesobius antipodum*, only half of the individuals harboured parasites.

**Table 2.** Taxonomic identities, prevalence (P), mean abundance (MA) and abundance range (R) of grenadier parasites recovered from the Chatham Rise

	CMA		CFA		BJA		JAV		CBI		CBO		CSU		CAS	
	(*n* = 3)		(*n* = 5)		(*n* = 12)		(*n* = 4)		(*n* = 6)		(*n* = 8)		(*n* = 7)		(*n* = 7)	
		MA		MA		MA		MA		MA		MA		MA		MA
	P	(R)	P	(R)	P	(R)	P	(R)	P	(R)	P	(R)	P	(R)	P	(R)
DIGENEA																
*Derogenes* sp._(A)_	–	–	–	–	–	–	–	–	–	–	12.5	0.13	–	–	–	–
												(0–1)				
Digenea gen sp._(A)_	–	–	–	–	–	–	25	0.25	–	–	–	–	–	–	–	–
								(0–1)								
*Dinosoma synaphobranchi*_(A)_	–	–	–	–	–	–	–	–	–	–	–	–	71.4	27*	–	–
														(0–77)*		
*Genolinea laticauda*_(A)_	–	–	40	0.4	–	–	–	–	–	–	–	–	–	–	14.3	0.43
				(0–1)												(0–3)
*Glomericirrus macrouri*_(A)_	–	–	80	1.8	–	–	–	–	–	–	–	–	42.9	0.14	–	–
				(0–5)										(0–1)		
*Gonocerca* sp._(A)_	–	–	–	–	–	–	–	–	–	–	–	–	–	–	14.3	0.14
																(0–1)
Hemiuridae gen sp._(A)_	–	–	–	–	–	–	–	–	16.7	0.17	25	6.25	–	–	–	–
										(0–1)		(0–49)				
Lecithochiriinae gen sp._(A)_	–	–	40	1	–	–	–	–	50	0.67	–	–	–	–	–	–
				(0–3)						(0–2)						
Lecithasterinae gen sp._(A)_	–	–	–	–	–	–	–	–	–	–	–	–	14.3	0.29	–	–
														(0–2)		
*Lepidapedon blairi*_(A)_	–	–	60	1.4	–	–	–	–	–	–	100	6.38	–	–	42.9	0.57
				(0–5)								(0–29)				(0–4)
*Lepidapedon* sp. 1_(A&L)_	100	17.33	–	–	–	–	–	–	–	–	–	–	–	–	–	–
		(1–28)														
*Lepidapedon* sp. 2_(A)_	–	–	–	–	–	–	–	–	–	–	37.5	13.25*	–	–	42.9	8.71*
												(0–50)*				(0–34)*
*Prodistomum* sp._(A)_	–	–	25	0.5	–	–	–	–	–	–	–	–	–	–	–	–
				(0–2)												
*Tellervotrema* sp._(A)_	–	–	50	0.5	–	–	–	–	–	–	–	–	–	–	–	–
				(0–1)												
CESTODA																
*Acanthobothrium filicolle*	–	–	–	–	–	–	25	12.5*	–	–	–	–	–	–	–	–
								(0–50)*								
Bothriocephalidea gen sp._(A)_	–	–	–	–	–	–	–	–	–	–	–	–	14.3	0.29	–	–
														(0–2)		
*Calyptrobothrium* sp.	100	16.67	–	–	–	–	50	–*	–	–	–	–	–	–	–	–
		(14–21)														
*Clestobothrium* sp.	–	–	–	–	–	–	25	–*	–	–	–	–	–	–	–	–
*Clistobothrium* sp.	–	–	–	–	8.3	0.08	–	–	–	–	–	–	–	–	–	–
						(0–1)										
*Grillotia* sp. 1	–	–	–	–	–	–	–	–	–	–	87.5	8.63	–	–	100	2.14
												(0–15)				(0–4)
*Grillotia* sp. 2	33.3	0.33	–	–	–	–	–	–	16.7	0.17	75	1.13	–	–	–	–
		(0–1)								(0–1)		(0–4)				
*Hepatoxylon trichiurid*	–	–	–	–	–	–	–	–	50	0.67	37.5	0.75	–	–	–	–
										(0–2)		(0–3)				
*Phyllobothrium* sp.	–	–	–	–	–	–	25	–*	–	–	–	–	–	–	–	–
*Trilocularia* sp.	–	–	–	–	–	–	50	0.25	–	–	–	–	–	–	–	–
								(0–1)								
*Yamaguticestus squali*	–	–	–	–	–	–	25	0.75	–	–	–	–	–	–	–	–
								(0–3)								
NEMATODA																
*Anisakis simplex* s.l. sp. A &sp. B*	–	–	20	–	41.7	–	25	–	83.3	–	37.5	–	42.9	–	57.1	–
Anticomidae gen sp	–	–	20	–	–	–	–	–	–	–	–	–	–	–	–	–
Ascarididae gen sp.	–	–	–	–	–	–	–	–	16.7	–	12.5	–	–	–	14.3	–
Cucullaninae gen sp.	–	–	–	–	–	–	50	–	–	–	–	–	–	–	–	–
*Hysterothylacium* sp._(A&L?)_	–	–	–	–	8.3	–	25	–	–	–	12.5	–	–	–	28.6	–
Leptosomatidae gen sp.	–	–	20	–	–	–	–	–	–	–	–	–	–	–	–	–
*Neoascarophis* sp.	–	–	–	–	–	–	–	–	–	–	–	–	–	–	42.9	–
*Neoterranova* sp._(A?)_	–	–	–	–	–	–	25	–	50	–	–	–	–	–	–	–
Spirumorpha gen sp.	33.3	–	40	–	–	–	–	–	–	–	–	–	–	–	–	–

Adult stage (A) parasites are indicated following their names (A&L indicates the presence of both adult and larval stage). Data with asterisk have been adjusted due to difficulty to distinguish morphologically similar parasites from the same hosts: MA and range of *Dinosoma synaphobranchi* include both *D. synaphobranchi* and *Glomericirrus macrouri*; MA and range of *Lepidapedon* sp. 2 include both *Lepidapedon blairi* and *Lepidapedon* sp. 2; MA and range of *Acanthobothrium fillicolle* also include *Clestobothrium* sp., *Calyptrobothrium* sp. and *Phyllobothrium* sp.; MA and range of *Calyptrobothrium* sp. for JAV include both *Calyptrobothrium* sp. and *Trilocularia* sp.. CMA: *Coelorinchus matamua*; CFA: *C. fasciatus*; CBI: *C. biclinozonalis*; CBO: *C. bollonsi*; CAS: *C. aspercephalus*; BJA: *Medobius antipodum*; JAV: *Lepidorhynchus denticulatus*; CSU: *Coryphaenoides subserrulatus*. All digenean species (except for Digenea gen sp.) have been identified by both genetics and morphology, while all cestode and nematode species have been determined by genetics only.

More than half (*n* = 555) of the parasites were digeneans, comprising a total of 14 species, which made it the most species-rich and common parasite group in this study. The most prevalent digenean was *Lepidapedon blairi,* which infected *Coelorinchus fasciatus*, *C. bollonsi* and *C. aspercephalus*. A second, cryptic species, *Lepidapedon* sp. 2, detected by DNA sequence, was also found to infect *C. bollonsi* and *C. aspercephalus*. The second most abundant parasites were cestodes (*n* = 262 individuals), represented by 11 species. The most prevalent cestode was *Grillotia* sp. 1, which was found in *C. bollonsi* and *C. aspercephalus*. Nematodes were the least abundant helminths (*n* = 167 individuals), constituting 10 species in total. *Anisakis* spp., including *Anisakis simplex* sensu lato (s.l) sp. A and *Anisakis simplex* s.l sp. B, were the most prevalent nematodes in this study, occurring in all species of grenadiers except for *C. matamua*. The high prevalence of *Anisakis* was mostly due to *A. simplex* s.l sp. A, since this species was found in 6 species of grenadiers, while *Anisakis simplex* s.l sp. B was only found in *Coryphaenoides subserrulatus*. However, because not all *Anisakis* nematodes were identified molecularly, whether *A. simplex* s.l sp. B only infects *C. subserrulatus* is unknown.

**Fig. 2.**
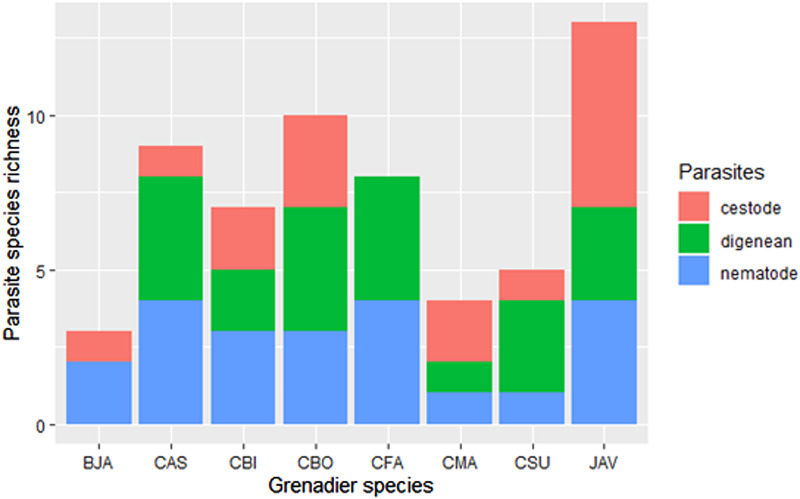
Parasite species richness among all 8 grenadier species – CMA: *Coelorinchus matamua*; CFA: *C. fasciatus*; CBI: *C. biclinozonalis*; CBO: *C. bollonsi*; CAS: *C. aspercephalus*; BJA: *Medobius antipodum*; JAV: *Lepidorhynchus denticulatus*; CSU: *Coryphaenoides subserrulatus*.

**Fig. 3.**
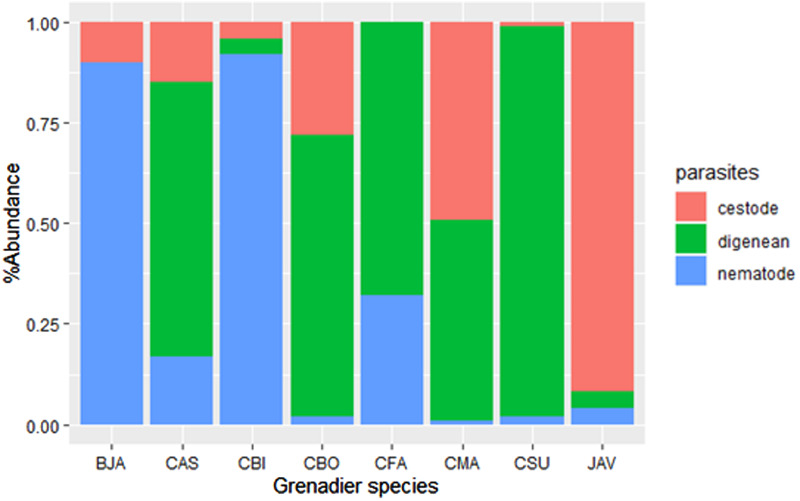
Percentage abundance of digeneans, cestodes, and nematodes in all 8 grenadier species – CMA: *Coelorinchus matamua*; CFA: *C. fasciatus*; CBI: *C. biclinozonalis*; CBO: *C. bollonsi*; CAS: *C. aspercephalus*; BJA: *Medobius antipodum*; JAV: *Lepidorhynchus denticulatus*; CSU: *Coryphaenoides subserrulatus*.

While most digeneans collected were adults, the majority of cestodes were larval stages, with only 2 adults found from a single *C. subserrulatus* host. Like cestodes, most nematodes were larvae as well. All grenadier species harboured nematodes, while no trematode was found in *M. antipodum*, and no cestode was found in *Coelorinchus fasciatus*. The highest parasite species richness was found in *Lepidorhynchus denticulatus* (Fig. 2); more than a quarter of species in this host were cestodes, with their mean abundance also being the highest (Fig. 3). Four out of five *Coelorinchus* host species hosted high digenean species richness and prevalence, although *Coryphaenoides subserrulatus* harboured the highest mean abundance of digeneans (Figs 2 and 3). *Coelorinchus bollonsi* had the highest mean abundance of parasites in this study, as well as the second highest parasite species richness and highest digenean species richness (Figs 2 and 3).

### Parasite assemblage visualization

**Fig. 4.**
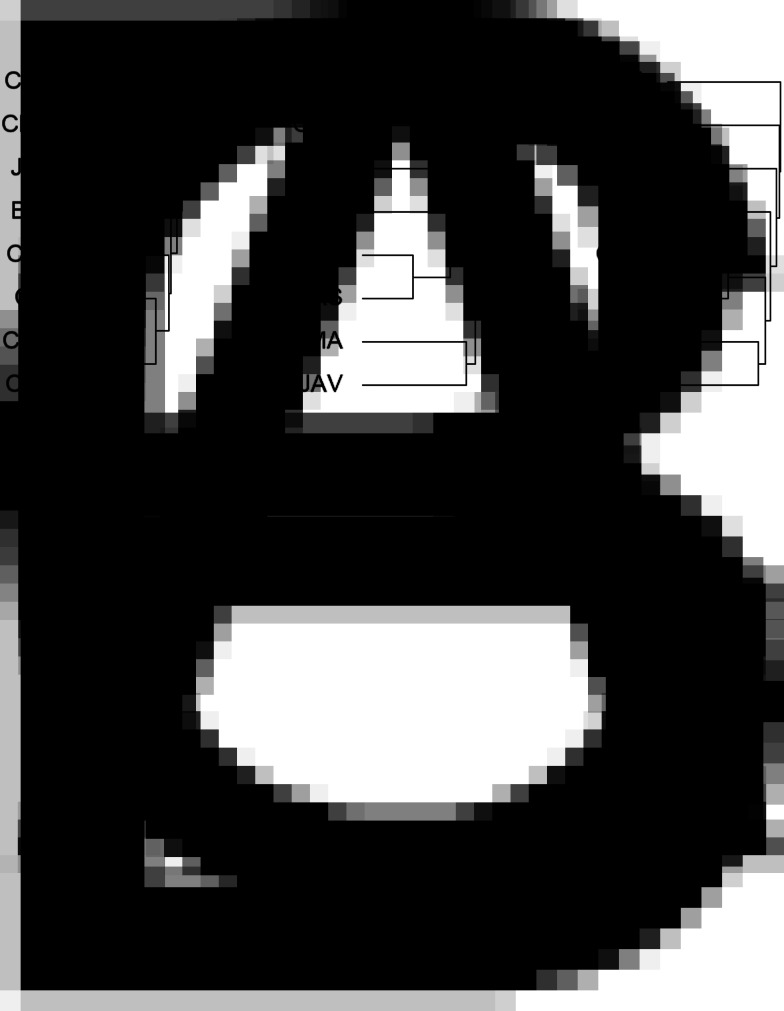
Dendrograms showing the cluster patterns produced by Hierarchical Agglomerative Clustering (HAC) analysis for the parasite assemblages among 8 grenadier species, computed using component community level parasite community descriptors (A) presence/absence; (B) prevalence; (C) mean abundance of each parasite species. CMA: *Coelorinchus matamua* (*n* = 3); CFA: *C. fasciatus* (*n* = 5); CBI: *C. biclinozonalis* (*n* = 6); CBO: *C. bollonsi* (*n* = 8); CAS: *C. aspercephalus* (*n* = 7); BJA: *Medobius antipodum* (*n* = 6); JAV: *Lepidorhynchus denticulatus* (*n* = 4); CSU: *Coryphaenoides subserrulatus* (*n* = 7). Each branch represents 1 grenadier species.

**Fig. 5.**
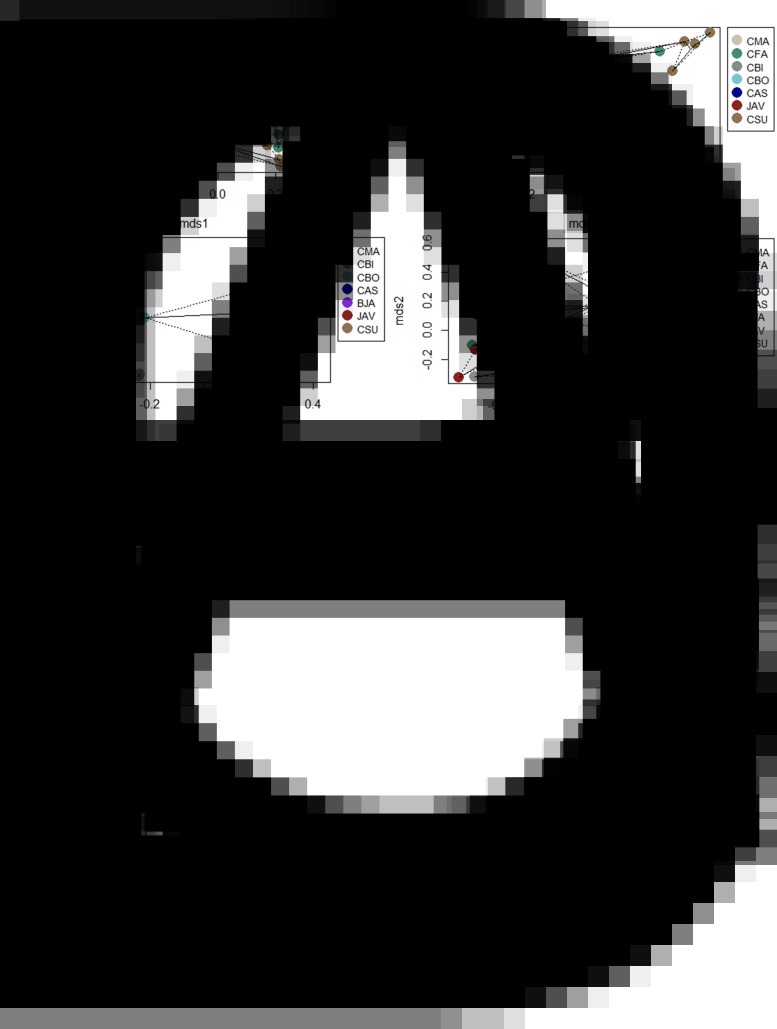
Classical multidimensional scaling (MDS) plot for species presence/absence of: (A) all parasites; (B) digeneans; (C) cestodes; (D) nematodes at Infracommunity level among 8 grenadier species – CMA: *Coelorinchus matamua* (*n* = 3); CFA: *C. fasciatus* (*n* = 5); CBI: *C. biclinozonalis* (*n* = 6); CBO: *C. bollonsi* (*n* = 8); CAS: *C. aspercephalus* (*n* = 7); BJA: *Medobius antipodum* (*n* = 6); JAV: *Lepidorhynchus denticulatus* (*n* = 4); CSU: *Coryphaenoides subserrulatus* (*n* = 7). Each dot represents a grenadier host individual, with host individuals of the same species connected by solid and broken lines. Stress level for each plot is <0.001.

In general, dendrograms of parasite presence/absence (Fig. 4A), prevalence (Fig. 4B) and mean abundance (Fig. 4C) at the component community level showed that except for *C. matamua*, 4 other species of *Coelorinchus* grenadiers tended to aggregate together to form a loose cluster structure. A similar pattern emerged in the dendrogram produced by both digenean and cestode presence/absence data but was not seen in the dendrogram produced by nematode data (Supplementary material, Fig S1). The *Coelorinchus* cluster was not obvious in the MDS plots and dendrograms at the infracommunity level, but grenadier hosts from 2 species of *Coelorinchus*, *C. bollonsi* and *C. aspercephalus* appeared to aggregate in all the MDS plots and dendrograms at the infracommunity level except for the analysis including nematode data only (Fig. 5, Supplementary material, Fig S2–4).

### Parasite assemblage analysis at the infracommunity level

The results of PERMANOVA analyses on both presence/absence and total abundance of parasites among 46 grenadier individuals (excluding 6 *M. antipodum* individuals that weren't infected by any parasite) showed that each grenadier species had different parasite assemblages (presence/absence: *F*_1,7_ = 5.23, *P*_(perm)_ = 0.001; total abundance: *F*_1,7_ = 5.45, *P*_(perm)_ = 0.001). PERMANOVA model checking by PERMDISP2 showed that there was no difference in multivariate dispersions between different grenadier species for both presence/absence (ANOVA: *F*_1,7_ = 1.61, *P* = 0.16 PERMUTEST: *F*_1,7_ = 1.61, *P*_(perm)_ = 0.16) and total abundance (ANOVA: *F*_1,7_ = 1.67, *P* = 0.15 PERMUTEST: *F*_1,7_ = 1.67, *P*_(perm)=_0.14) data. Detailed results for pairwise PERMANOVA are given in Supplementary material.

### Species richness as a function of fish length and fish species

The results of GLM showed that fish length had no relationship with parasites species richness (GLM with Poisson family, Likelihood Ratio/Chisquare = 0.01, Df = 1, *P* = 0.91), however, different grenadier species had different parasite species richness (GLM with Poisson family, Likelihood Ratio/Chisquare = 41.48, Df = 7, *P* < 0.001). Detailed AIC model selection and GLM results are given in Supplementary material.

### Distance decay of similarity in community composition

**Fig. 6.**
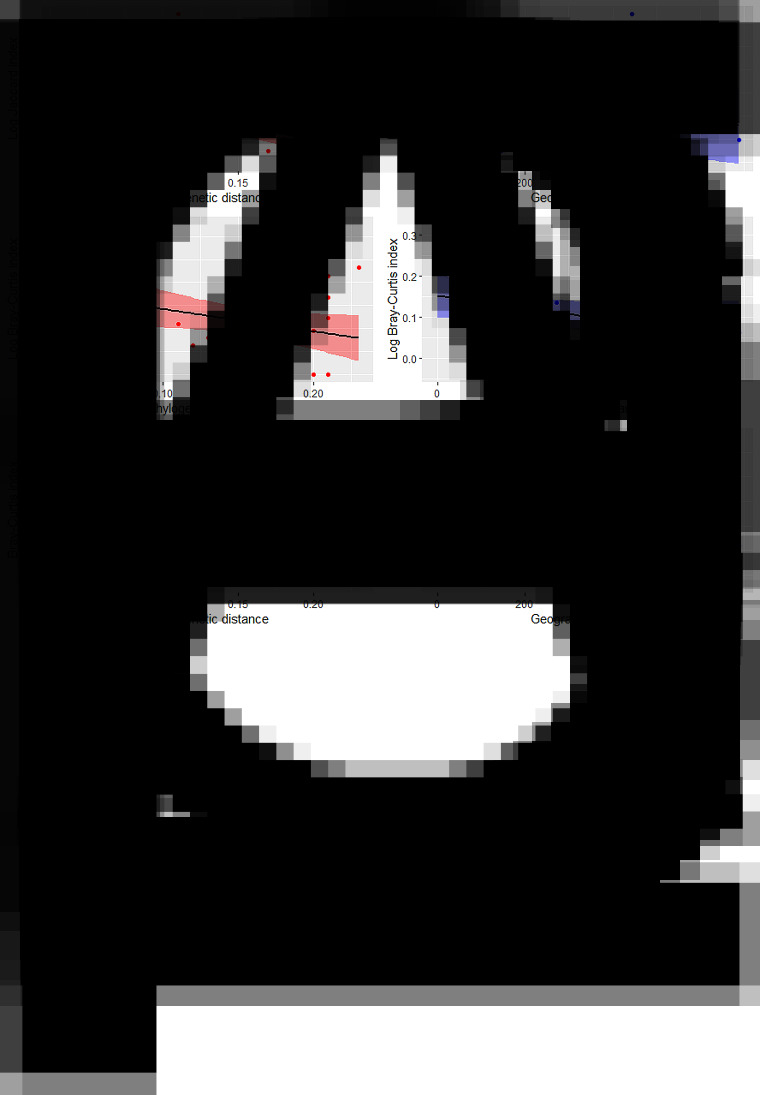
Scatter plots for parasite assemblages similarity: (A, B) Log-transformed Jaccard similarity based on presence/absence data; (C, D) Log-transformed Bray–Curtis similarity based on prevalence; (E, F) Bray–Curtis similarity based on mean abundance against pairwise phylogenetic distances between grenadier hosts (left) and geographic distance between trawling localities (right). The 95% confidence interval is indicated by red and purple shaded areas.

Multiple linear regression modelling showed that log-transformed pairwise Jaccard similarity values based on the presence/absence data decreased with increasing phylogenetic distance (*R*^2^ = 0.2, *P* = 0.01, effect size = 0.25, Fig. 6A) among species of grenadier hosts but were not affected by geographic distance (*R*^2^ = 0.2, *P* = 0.94, effect size<0.001) among different trawling sites (Fig. 6B). However, similarity values computed using parasite prevalence data decreased with both increasing phylogenetic distance (*R*^2^ = 0.36, *P* = 0.01, effect size = 0.25, Fig. 6C) among fish hosts and increasing geographic distance (*R*^2^ = 0.36, *P* = 0.002, effect size = 0.32, Fig. 6D) (results here should be treated with caution considering the high number of outliers). In parallel, similarity values based on mean abundance data also showed a decrease with both increasing phylogenetic distance (*R*^2^ = 0.47, *P* < 0.001, effect size = 0.43, Fig. 6E) and geographic distance (*R*^2^ = 0.47, *P* = 0.002, effect size = 0.33, Fig. 6F). Linear regressions based on permutations supported these results (data not shown).

The distribution of data points in all phylogenetic distance (i.e. the inverse of phylogenetic relatedness) figures (Fig. 6A, C, E) suggest that very closely related grenadier species tend to have more similar parasite assemblages than expected, with the similarity dropping below that predicted by the model as the phylogenetic distance increased (pairwise phylogenetic distances presented in Supplementary material Table S3). Among distantly related fish species, parasite communities were either more similar or less similar than model predictions.

In contrast to the phylogenetic relatedness scatter plots, the distribution of data points in geographical distance (pairwise distance data presented in Supplementary material Table S4) scatter plots showed different patterns. In Fig. 6D and F, the parasite assemblages of many pairs of grenadier species became less similar than predicted by the model as the distance between sample sites increased. Beyond a distance of about 400 km, grenadier species tended to harbour more similar parasite faunas than predicted by the model.

### Species accumulation curve

The cumulative number of parasite species collected from 46 grenadier hosts in this study appeared to level off as more individual hosts were examined, showing signs of reaching an asymptote. This suggests most parasite species have been sampled, except for perhaps some highly host-specific species with low prevalence (Supplementary material, Fig. S5). However, since the single accumulation curve was produced by pooling all infected grenadier individuals across all 8 species, this finding must be interpreted with caution.

## Discussion

Logistical challenges associated with access to deep-sea organisms explain the very limited number of parasitological studies on deep-sea fishes compared with commercially important fish or those from shallow coastal regions. To date, there have been very few comprehensive fish parasite studies in NZ waters (Manter, 1954; Brunsdon, 1956; Hine *et al*., 2000). Only 4 digenean species for *Coelorinchus australis* were recorded by Manter (1954), and only a handful of known endoparasites are listed in Hine *et al*. (2000) for 16 species of NZ grenadiers. Meanwhile, despite the growing realization that host phylogenetic relatedness and geographic distance are key determinants of differences in parasite assemblages (Pérez-del-Olmo *et al*., 2009; Poulin, 2010), their effects have never been investigated in deep-sea systems. This represents a knowledge gap in our understanding of the factors that influence community assembly processes in the deep-sea.

This is the first study to (1) investigate the diversity and species composition of parasites of 8 species of NZ grenadiers from the Chatham Rise and (2) evaluate the effects of phylogeny and geographic distances on interspecific patterns of parasite community similarity. Despite different species harbouring different parasite assemblages, the parasite community structure of 4 out of 5 *Coelorinchus* species showed some degree of similarity by sharing several species of digeneans, cestodes and nematodes. The distance-decay analysis showed that the presence/absence similarity of parasites was only affected by the phylogenetic relatedness among grenadier species, whereas both phylogenetic distance among grenadier species and geographic distance between sample locations influenced the similarity of parasite communities based on prevalence (although the latter finding should be treated with caution) and mean abundance.

The small and uneven sample size for each grenadier species in this study, the fact that each fish species has been obtained at a different trawling location with a different depth range, and the geographical distance among localities of capture being latitudinally and longitudinally narrow (43°S–44.30ʹS–177°00ʹE–174°30ʹW), warrant some caution regarding the interpretation of the results. Nevertheless, our results are comparable to the few other studies of deep-sea parasites and also parasite assemblage studies in various systems (addressed below), ultimately supporting the main finding that the distance-decay of community similarity found in other systems might also apply to the deep-sea.

### Parasite assemblages of NZ grenadiers and host phylogenetic relatedness

Putting the phylogenetic relatedness into a distance-decay framework enables a quantitative approach to test the correlation between host phylogeny and the proportional similarity of their parasite assemblages. This approach has only been used by Seifertova *et al*. (2008) and Poulin (2010), therefore, the universality of this phenomenon remains to be tested. Compared with the proportion of variation in parasite community similarity explained by phylogenetic relatedness among 45 species of freshwater fish in Poulin (2010), phylogenetic distance among grenadiers explained a higher proportion of variance (*R*^2^ = 0.2). The present result suggests that phylogenetic relatedness of NZ grenadiers shapes the composition of their parasite assemblages.

A good example involves the 2 closely related *Coelorinchus* species, *C. bollonsi* and *C. aspercephalus*, which have similar parasite assemblages. The fact that both species (except for 1 *C. bollonsi* individual – CBO1 071, caught at a different site) were caught in the same site suggests they have similar habitat requirements. Stevens and Dunn (2011) also showed that they both are predominantly benthic feeders, but with *C. bollonsi* feeding on in- and epi-faunal polychaetes and *C. aspercephalus* on epi-faunal crustaceans. Since the ecology and phylogeny of species are not independent, due to phylogenetic niche conservatism (Peterson *et al*., 1999), the similar habitat and feeding ecology of these 2 *Coelorinchus* species possibly results from their phylogenetic relatedness. Thus, from a host–parasite point of view, closely related hosts should have similar immunological and physiological properties, feeding behaviour and live in similar habitats, ultimately resulting in similar parasite communities.

Alternatively, hosts that are not closely related can also be infected by the same parasite taxa by having the same diet (or feeding on a range of crustaceans that are intermediate hosts of the same digenean species) in the same environment. Both *Coelorinchus fasciatus* and *Coryphaenoides subserrulatus* harboured *Glomericirrus macrouri*, providing an example of this scenario. The fact that both species were infected by a shared digenean species suggests a potential overlap in their diet, although they belong to different genera. This is one of the reasons why phylogenetic relatedness only explains a small proportion of the differences in their parasite assemblages. Grenadiers are commonly considered as generalist feeders (Mauchline and Gordon, 1984), but the fact that they can have different assemblages of parasites suggests some degree of specialization in their feeding behaviour.

For deep-sea fish parasites, host feeding ecology is believed to be the primary driver of their parasite assemblages, not their phylogeny (Campbell, 1983; Palm and Klimpel, 2008). Our study suggests that phylogenetic affinities among grenadier hosts increases their chance of harbouring similar parasites. This does not diminish the well-recognized significance of feeding ecology, since host phylogeny shapes their ecological characteristics. Thus, phylogeny may be the ultimate evolutionary driver of parasite community composition in grenadiers.

### Parasite assemblages of NZ grenadiers as a function of geographic distance

Diminishing similarity in species composition with increasing geographical distance is a universal phenomenon observed in multiple systems (Nekola and White, 1999), including host–parasite systems (Poulin, 2003). However, the total number of studies of this pattern remains scarce, especially for marine fish parasites (Oliva and Gonzalez, 2005; Pérez-del-Olmo *et al*., 2009; Braicovich *et al*., 2017). To date, only the parasite assemblages of coastal marine fish have been investigated for geographical distance decay patterns. We tested this phenomenon in a localized deep-sea fish meta-community consisting of multiple sympatric fish species belonging to the same family. Surprisingly, our analysis found that although geographic distance had no impact on parasite community similarity when based on presence/absence data, the expected negative trend was observed when using data on the prevalence and mean abundance of parasites. This finding contrasts with some of the patterns found in coastal fish parasite studies (e.g. parasite assemblage of *Hippoglossina macrops* from the coast of Chile and southern Argentina, Oliva and Gonzalez, 2005), in which the similarity of parasite species composition did not decrease with increasing geographical distance. Pérez-del-Olmo *et al*. (2009) concluded that this is mainly caused by the absence of physical barriers in the open ocean, allowing the dispersal of parasites by their coastal fish hosts and the homogenization of parasite assemblages at a large geographical scale. In the deep sea, one of the biggest challenges is the scarcity of nutrients in the deep pelagic ocean caused by steep vertical gradients in abiotic and biotic factors (Rex and Etter, 2010). As a potential coping mechanism for an environment with limited energy, grenadiers might decrease their basic activity level to conserve energy. Their restricted movement within a localized area could ultimately set a dispersal limit for parasites, creating heterogeneous parasite assemblages with increasing geographic distance. However, our results must be taken with caution due to the limited sample sizes and small geographic area tested.

The overall similarity decay in parasite species composition may also reflect a change of host diet as the geographic distance between localities increases, due to spatial differences in the available invertebrate prey items. As the sediment conditions vary from sandy to muddy, the local invertebrate community along the Chatham Rise may change from predominantly crustaceans to echinoderms (McKnight and Probert, 1997). A similar situation may exist for the species composition of polychaetes, which appeared to be locally distinct along the Chatham Rise (Probert *et al*., 1996). The abundance of available prey items can also vary among sites, as the benthic biomass in southern parts of the Rise is higher than in northern sites (Nodder *et al*., 2003). All sample sites in this study were at least 100 km apart; it is thus unlikely that the same invertebrates would occur at all sites at similar abundances, causing the availability of intermediate hosts for parasites to vary among sampling sites.

Compared with the presence/absence of parasites, other population descriptors such as prevalence and mean abundance are thought to be more prone to local variation (Janovy *et al*., 1992). The results of the current study validate this statement, as prevalence and abundance data suggest an influence of geographical distance that was missed by presence/absence data. Interestingly, according to its effect size, geographical distance seems to have a larger effect on parasite similarity based on prevalence than phylogenetic relatedness, with the opposite for mean abundance, suggesting stronger evolutionary influence on abundance than on prevalence. Poulin (2006) found that the abundance of the same species of parasites stayed roughly constant among different local populations of parasites, but not their prevalence, which suggested that abundance is a species trait. This may explain the present results. Since abiotic factors affect the survival and transmission of free-living parasite larvae, the encounter of hosts with their parasites depends on external environmental conditions, which ultimately determine the number of hosts being infected (Pietrock and Marcogliese, 2003). Furthermore, biotic variables such as the diversity of prey items, also mediate the probability of an infected prey being ingested, and eventually affect prevalence (Poulin, 2006). On the other hand, abundance is more likely to be influenced by processes within fish hosts, such as density-dependent interactions among conspecific parasites (Shostak and Scott, 1993).

Overall, grenadiers from the Chatham Rise appeared to harbour a higher diversity of digeneans, cestodes and nematodes than previously thought. Predominantly deep-sea parasite taxa found in other geographical areas also occurred on the Chatham Rise. Though few parasites could be identified down to species level, the overall species identification effort showed most of the parasites harboured by NZ grenadiers are generalists, with the presence of few potentially host-specific *Lepidapedon* species in their *Coelorinchus* hosts, as seen in other deep-sea studies (Klimpel *et al*., 2009). Two possible new *Lepidapedon* species were also discovered, one of them assumed to be a cryptic sister species to *Lepidapedon blairi*. Improving knowledge of endoparasites infecting NZ grenadiers not only contributes to overall knowledge of parasite biodiversity in NZ (Bennett *et al*., 2021), but also advances our understanding of biodiversity and biology of deep-sea fishes. We recovered on average 4.4 parasite species per grenadier species. The species accumulation curve suggested that we achieved an adequate estimation of the parasite biodiversity of the Chatham Rise. Most importantly, the current study highlighted the significant effect of deep-sea host phylogeny in shaping their parasite assemblages, a factor previously underappreciated in studies of parasite communities in deep-sea systems. However, more extensive sampling at varying geographical scales will be needed to confirm this point, and to resolve the relative roles of host ecology and phylogeny in explaining parasite assemblages in deep-sea ecosystems.

## Supplementary material

For supplementary material accompanying this paper visit https://doi.org/10.1017/S0031182022001251. click here to view supplementary material
