# Supplementary material for: Decay of parasite community similarity with host phylogenetic and geographic distances among deep-sea fish (grenadiers)
Source: Parasitology. 2022 Aug 25;149(13):1737–48. doi: 10.1017/S0031182022001251 (PMC10090766; doi:10.1017/S0031182022001251)
Supplement: Supplementary file 1 [file S0031182022001251sup001.docx]

Supplementary Material

**
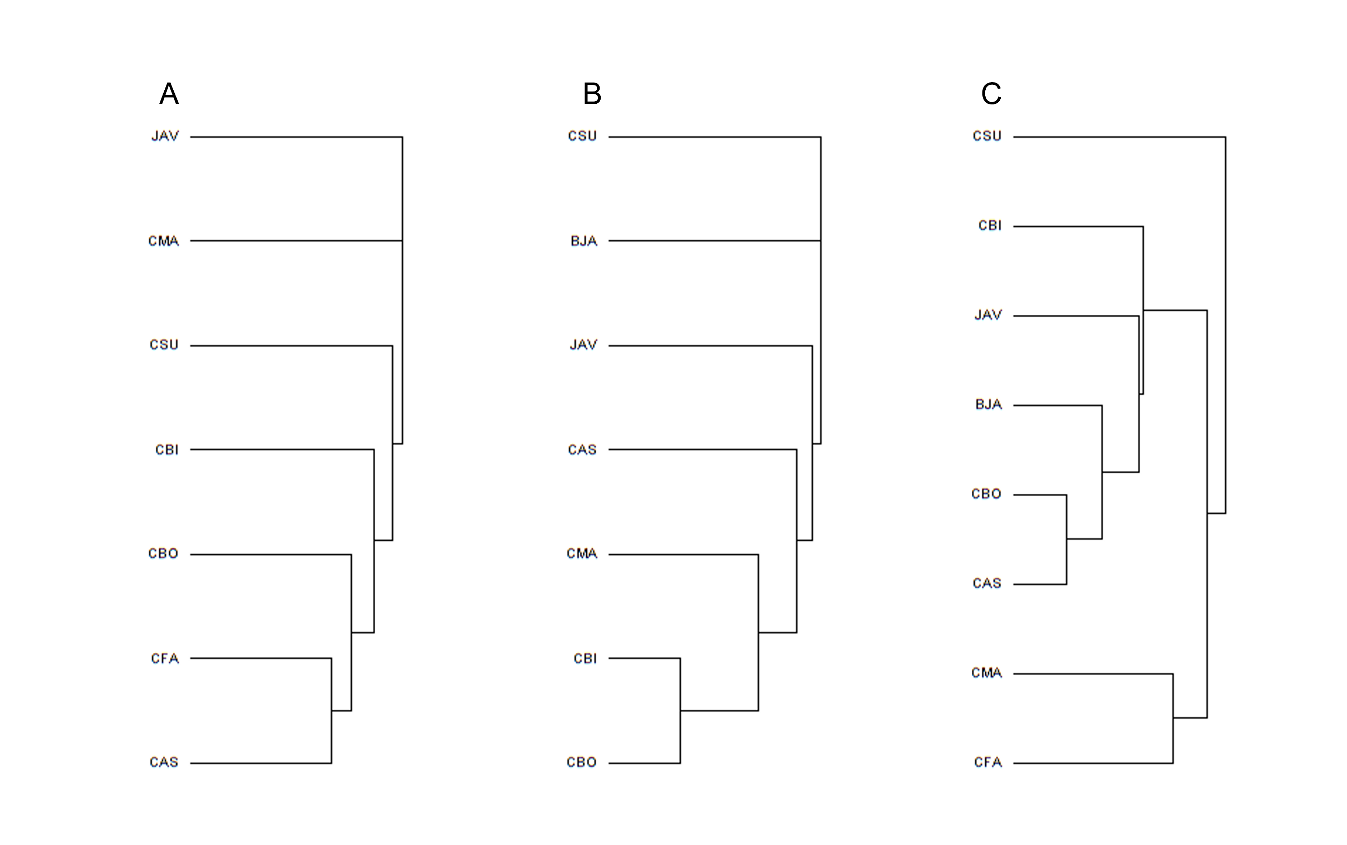
**

Figure S1. Dendrograms showing the cluster patterns produced by Hierarchical Agglomerative Clustering (HAC) analysis for species presence/absence of A: digeneans; B: cestodes; C: nematodes among 8 grenadier species-- CMA: Coelorinchus matamua (n=3); CFA: C. fasciatus (n=5); CBI: C. biclinozonalis (n=6); CBO: C. bollonsi (n=8); CAS: C. aspercephalus (n=7); BJA: Medobius antipodum (n=6); JAV: Lepidorhynchus denticulatus (n=4); CSU: Coryphaenoides subserrulatus (n=7), computed using component community level parasite data. Each branch represents one grenadier species.


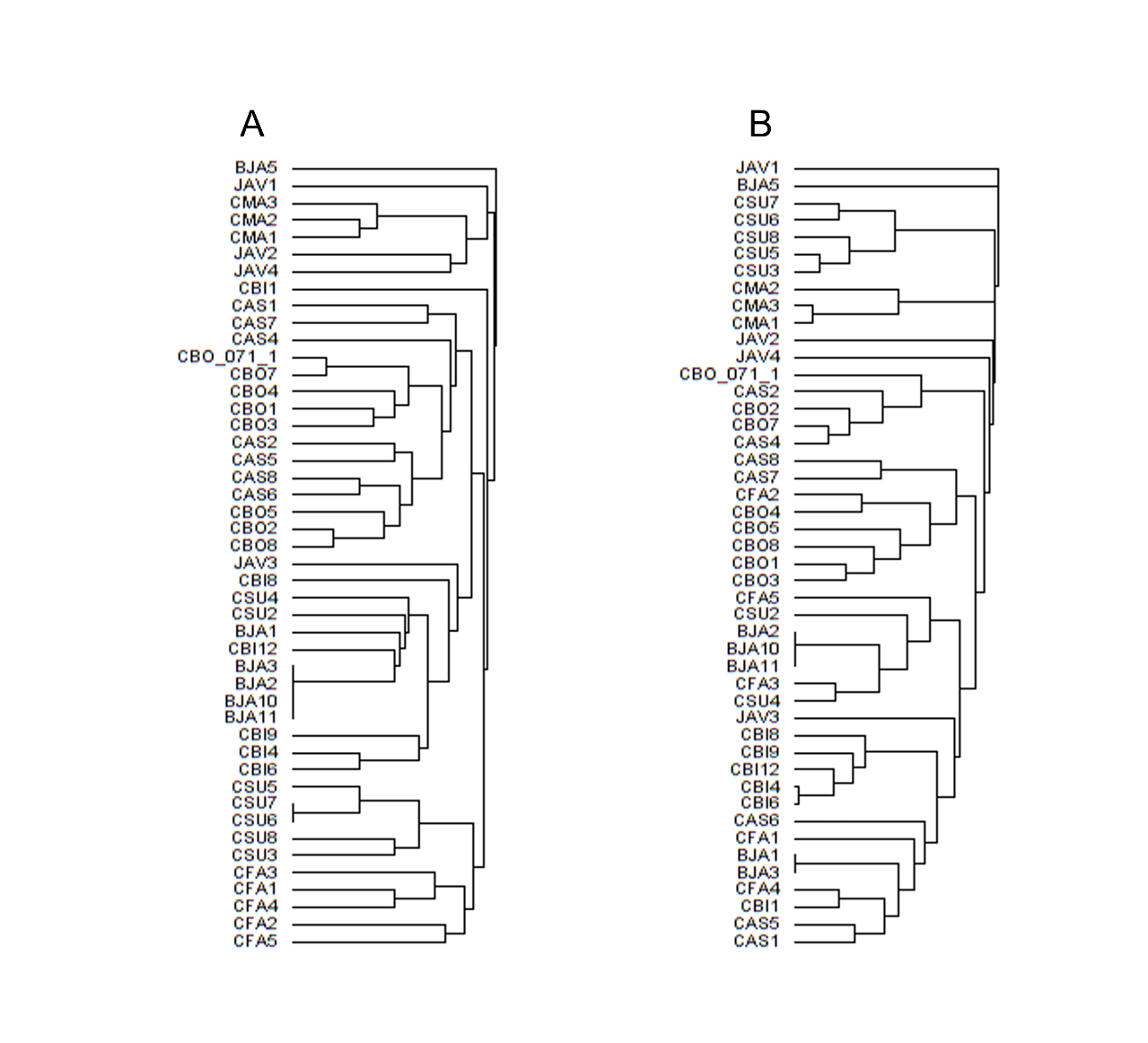


Figure S2. Dendrograms showing the cluster patterns produced by Hierarchical Agglomerative Clustering (HAC) analysis for parasite A: presence/absence; and B: total abundance at infra-community level among 8 grenadier species--- CMA: Coelorinchus matamua (n=3); CFA: C. fasciatus (n=5); CBI: C. biclinozonalis (n=6); CBO: C. bollonsi (n=8); CAS: C. aspercephalus (n=7); BJA: Medobius antipodum (n=6); JAV: Lepidorhynchus denticulatus (n=4); CSU: Coryphaenoides subserrulatus (n=7). Each branch represents a grenadier host individual.


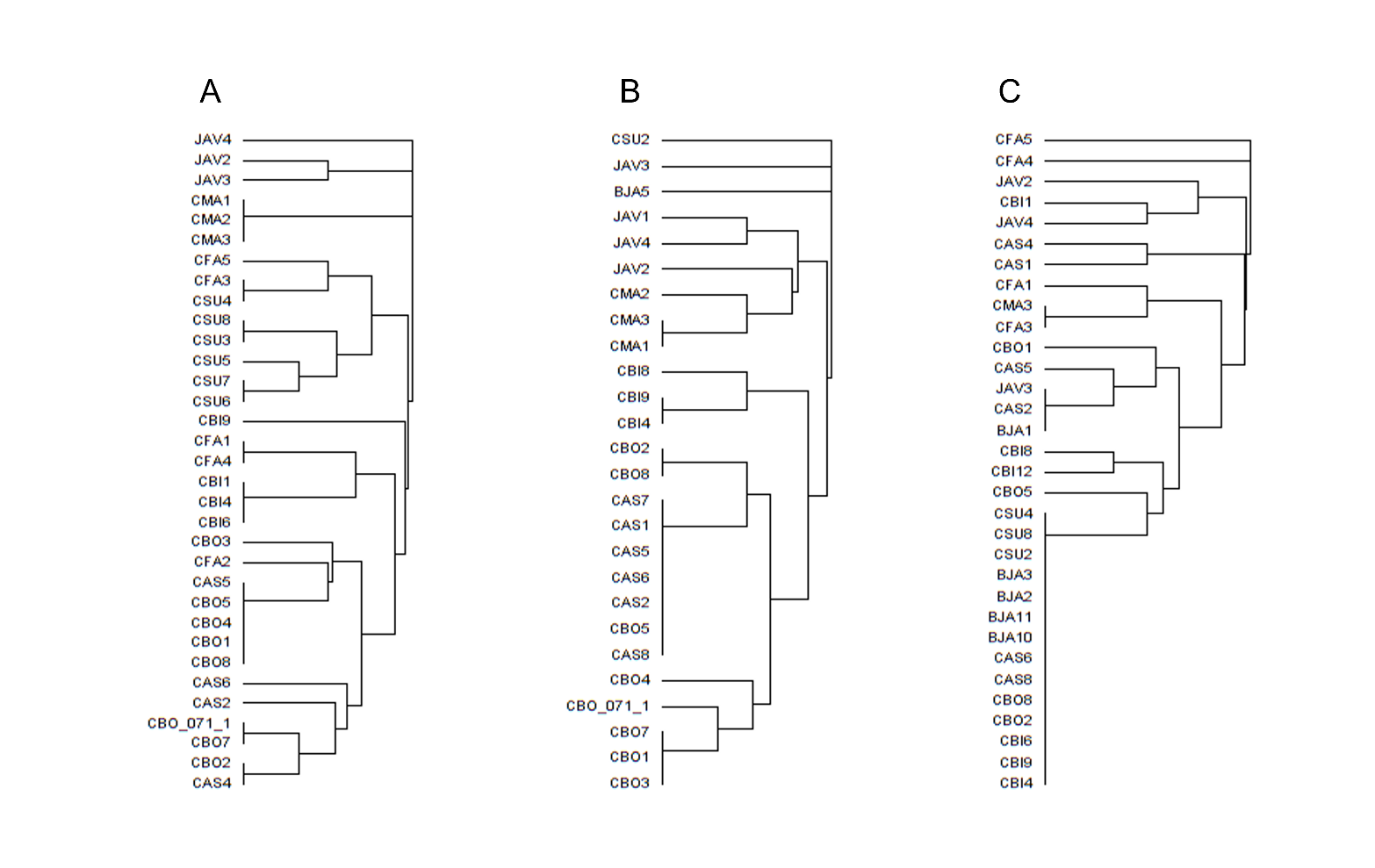


Figure S3. Dendrograms showing the cluster patterns produced by Hierarchical Agglomerative Clustering (HAC) analysis for species presence/absence of A: digeneans; B: cestodes; C: nematodes at infra-community level among 8 grenadier species--- CMA: Coelorinchus matamua (n=3); CFA: C. fasciatus (n=5); CBI: C. biclinozonalis (n=6); CBO: C. bollonsi (n=8); CAS: C. aspercephalus (n=7); BJA: Medobius antipodum (n=6); JAV: Lepidorhynchus denticulatus (n=4); CSU: Coryphaenoides subserrulatus (n=7). Each branch represents a grenadier host individual.


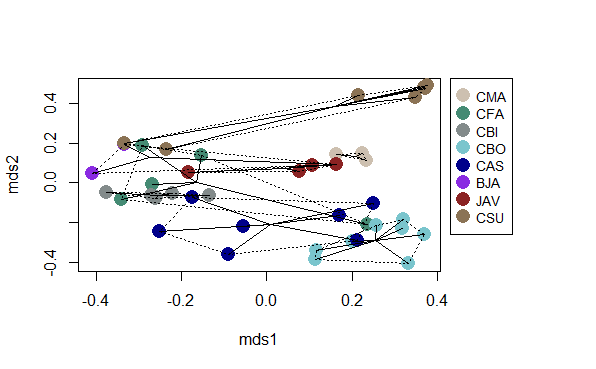


Figure S4. Classical multidimensional scaling (MDS) plot for parasite total abundance at infra-community level among 8 grenadier species--- CMA: Coelorinchus matamua (n=3); CFA: C. fasciatus (n=5); CBI: C. biclinozonalis (n=6); CBO: C. bollonsi (n=8); CAS: C. aspercephalus (n=7); BJA: Medobius antipodum (n=6); JAV: Lepidorhynchus denticulatus (n=4). CSU: Coryphaenoides subserrulatus (n=7). Each dot represents a grenadier host individual, with host individuals of the same species connected by solid and broken lines.


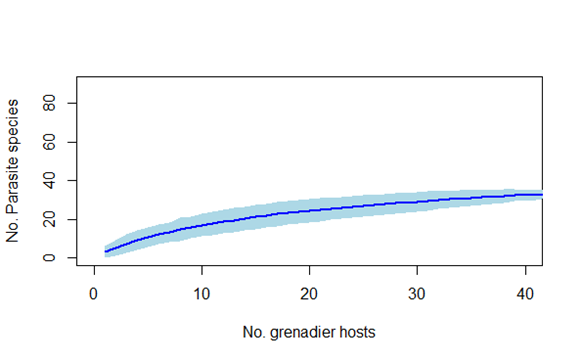


Figure S5. Species accumulation curve. Number of parasite species recovered plotted against the cumulative number of host individuals infected by parasites examined. The 95% confidence interval is indicated by the light blue area.

**Extra results**

*Parasite assemblage analysis at the infracommunity level*

The results of pairwise PERMANOVA on both presence/absence and total abundance of parasites among 46 grenadier individuals showed that only about a quarter of the pairwise comparisons among different grenadier species were significant or marginally significant, and with more differences between *Coelorinchus* species and species from other genera than among *Coelorinchus* species (Table S1-2). This result supported the *Coelorinchus* cluster revealed in the MDS plots and dendrograms at infra-community and component community levels.

*Species richness as a function of fish length and fish species—AIC model selection*

Model selection with AIC showed both GLMs had better fit with “poisson” distribution than “negative binomial” distribution (fish length: Poisson AIC: 165.62, negative binomial AIC: 166.54; fish species: Poisson AIC: 176.92, negative binomial AIC: 178.92).

Table S1. Pair-wise PERMANOVA result outputs on presence/absence of parasites at infra-community level among 8 grenadier species--- CMA: Coelorinchus matamua (n=3); CFA: C. fasciatus (n=5); CBI: C. biclinozonalis (n=6); CBO: C. bollonsi (n=8); CAS: C. aspercephalus (n=7); BJA: Medobius antipodum (n=6); JAV: Lepidorhynchus denticulatus (n=4); CSU: Coryphaenoides subserrulatus (n=7). Significant adjusted p values are in bold, adjusted p values close to 0.05 margin are underlined.


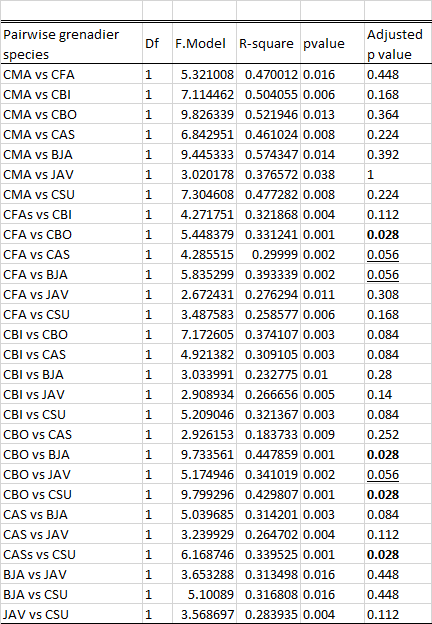


Table S2. Pair-wise PERMANOVA result outputs on total abundance of parasites at infra-community level among 8 grenadier species--- CMA: Coelorinchus matamua (n=3); CFA: C. fasciatus (n=5); CBI: C. biclinozonalis (n=6); CBO: C. bollonsi (n=8); CAS: C. aspercephalus (n=7); BJA: Medobius antipodum (n=6); JAV: Lepidorhynchus denticulatus (n=4); CSU: Coryphaenoides subserrulatus (n=7). Significant adjusted p values are in bold, adjusted p values close to 0.05 margin are underlined.


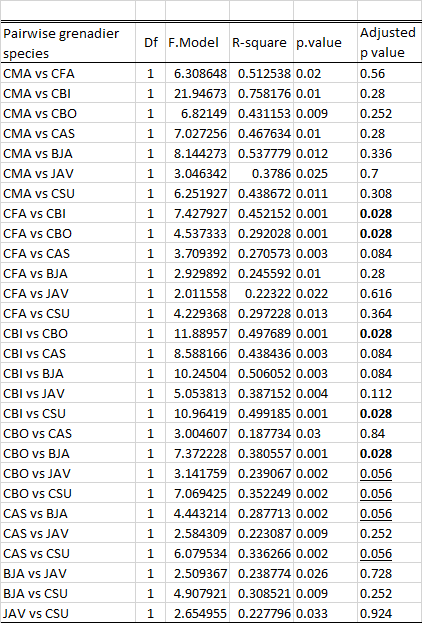


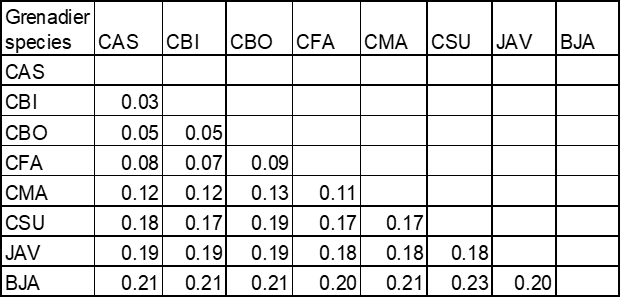
Table S3. Pair-wise phylogenetic distances between grenadier species in this study. Phylogenetic distance measured as the number of base pair differences per site between the cox1 sequences. CMA: Coelorinchus matamua; CFA: C. fasciatus; CBI: C. biclinozonalis; CBO: C. bollonsi; CAS: C. aspercephalus; BJA: Medobius antipodum; JAV: Lepidorhynchus denticulatus; CSU: Coryphaenoides subserrulatus.

Table S4. Pair-wise geographical distances (km) between sampling sites of grenadier species in this study. CMA: Coelorinchus matamua; CFA: C. fasciatus; CBI: C. biclinozonalis; CBO: C. bollonsi; CAS: C. aspercephalus; BJA: Medobius antipodum; JAV: Lepidorhynchus denticulatus; CSU: Coryphaenoides subserrulatus.

Table S5. BLAST results for 28S sequences of A) digeneans; B) cestodes; and 18S sequences for C) nematodes of grenadiers.

A)


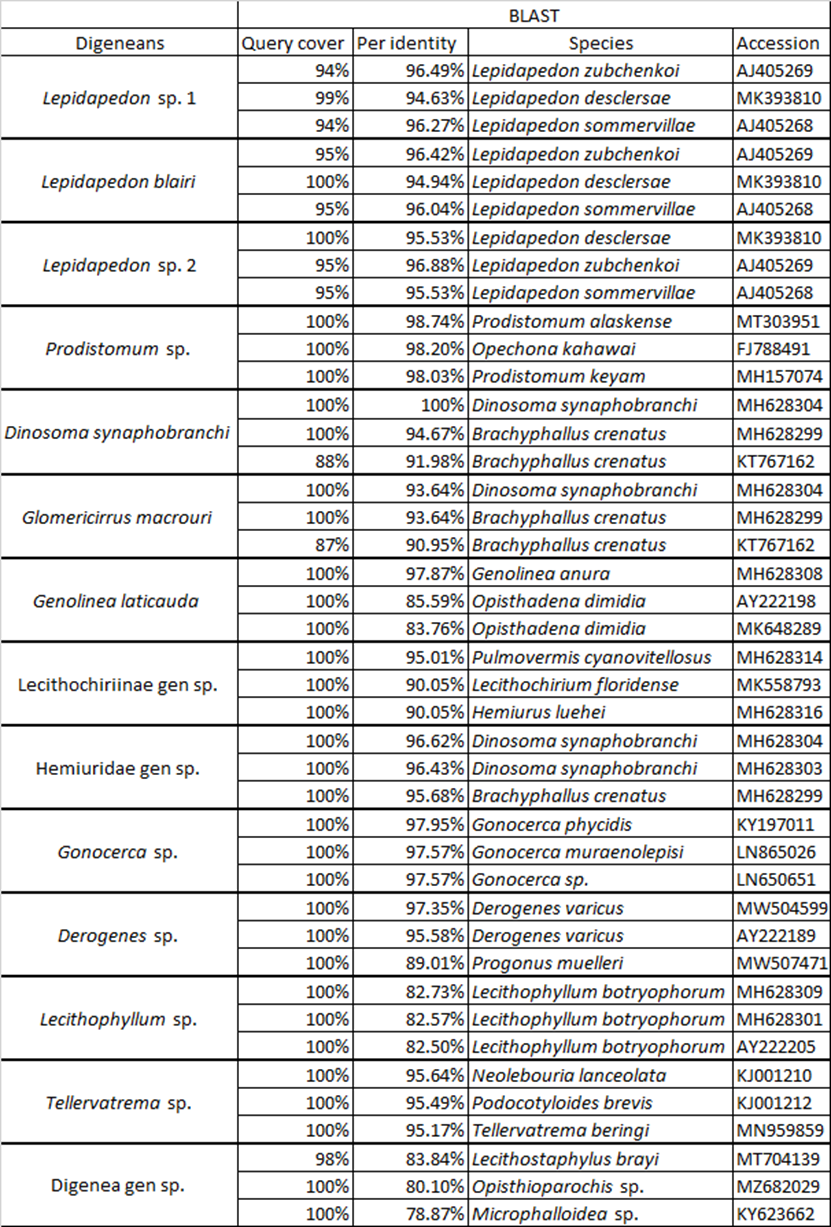


Table S5 continued

B)


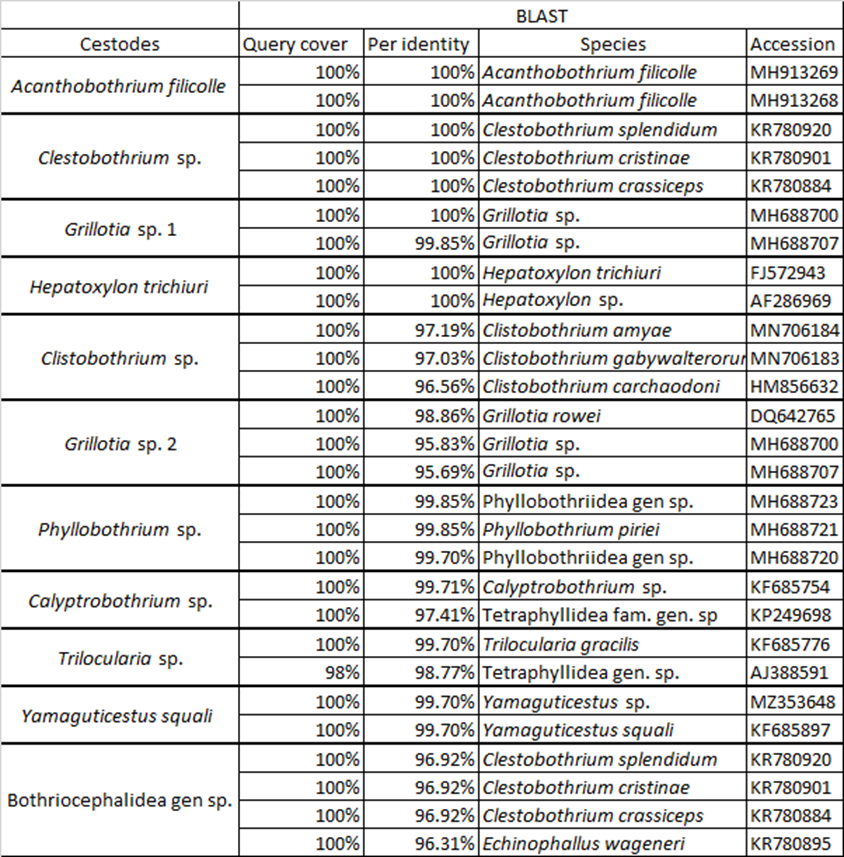


Table S5 continued

C)


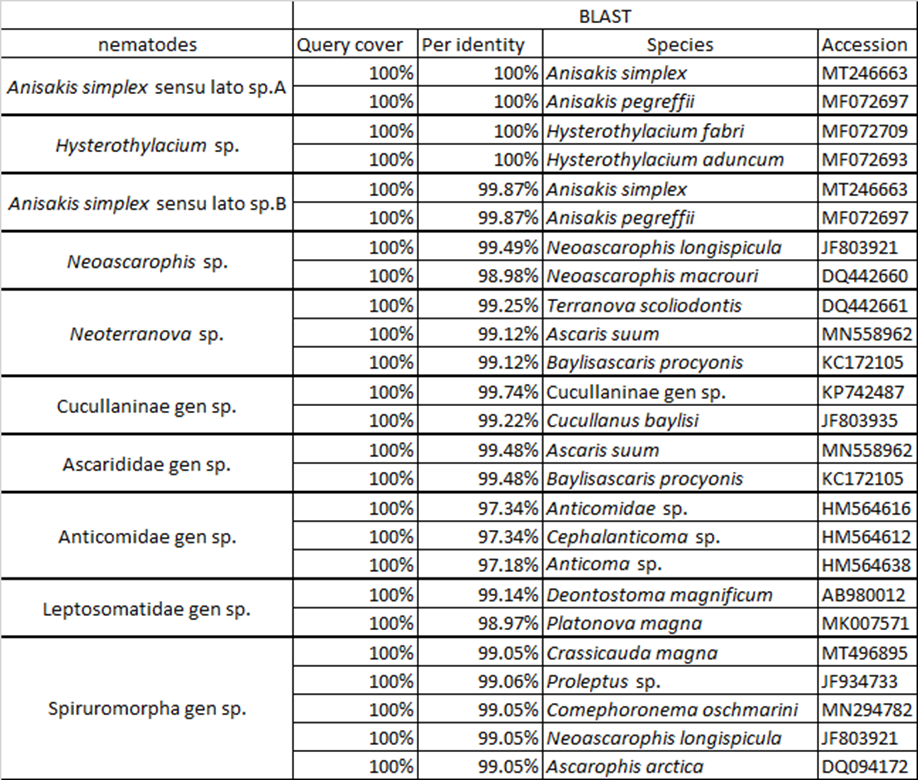


Table S6. BLAST results for grenadiers cox1 sequences.


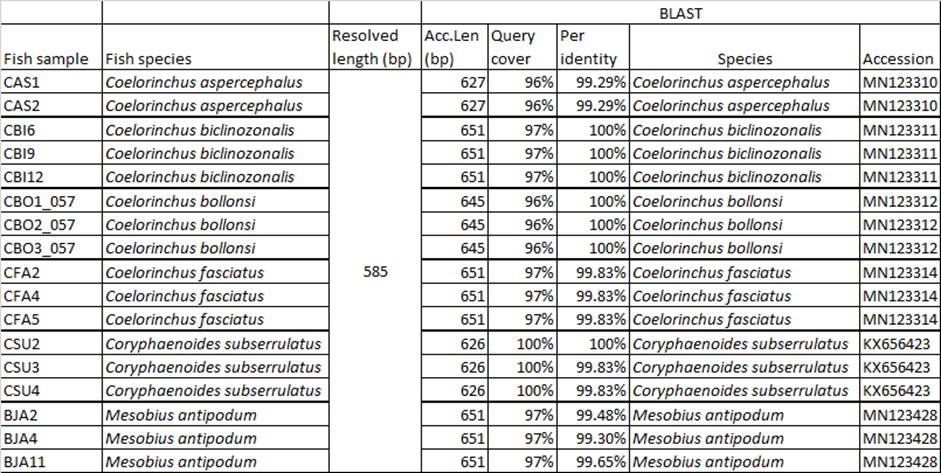


Table S7. Statistical results for two GLMs.

i)Predicator variable: Fish length; Response variable: parasite species richness


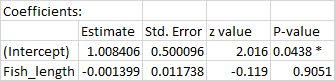

ii)Predictor variable: Fish species; Response variable: parasite species richness. CMA: *Coelorinchus matamua*; CFA: *C. fasciatus*; CBI: *C. biclinozonalis*; CBO: *C. bollonsi*; CAS: *C. aspercephalus*; JAV: *Lepidorhynchus denticulatus*; CSU: *Coryphaenoides subserrulatus*.
